# Analyzing SystemC Designs: SystemC Analysis Approaches for Varying Applications

**DOI:** 10.3390/s150510399

**Published:** 2015-05-04

**Authors:** Jannis Stoppe, Rolf Drechsler

**Affiliations:** 1 German Research Center for Artificial Intelligence DFKI, Research Department for Cyber-Physical Systems, Bibliothekstr. 1, Bremen D-28359, Germany; 2 University of Bremen, Group of Computer Architecture, Bibliothekstr. 1, Bremen D-28359, Germany, E-Mail: drechsler@uni-bremen.de

**Keywords:** SystemC, ESL, analysis, machine learning, parsers, AOP, hardware/software co-design

## Abstract

The complexity of hardware designs is still increasing according to Moore's law. With embedded systems being more and more intertwined and working together not only with each other, but also with their environments as cyber physical systems (CPSs), more streamlined development workflows are employed to handle the increasing complexity during a system's design phase. SystemC is a C++ library for the design of hardware/software systems, enabling the designer to quickly prototype, e.g., a distributed CPS without having to decide about particular implementation details (such as whether to implement a feature in hardware or in software) early in the design process. Thereby, this approach reduces the initial implementation's complexity by offering an abstract layer with which to build a working prototype. However, as SystemC is based on C++, analyzing designs becomes a difficult task due to the complex language features that are available to the designer. Several fundamentally different approaches for analyzing SystemC designs have been suggested. This work illustrates several different SystemC analysis approaches, including their specific advantages and shortcomings, allowing designers to pick the right tools to assist them with a specific problem during the design of a system using SystemC.

## 1. Introduction

With the ever-increasing complexity of hardware designs, their development process is facing the problem of the so-called design gap [1]: the ability to build complex systems grows much faster than the ability to design them. This specifically applies to embedded systems and, with them, also to compound embedded systems that focus on interactions with each other and their environment, so-called cyber-physical systems (CPSs). One way to approach this issue is to employ more streamlined development workflows to handle the increasing complexity during a system's design phase. This especially means that the designer's ability to quickly design a working prototype should be increased by reducing the complexity needed to build one.

SystemC is a C++ library that allows one to model hardware/software systems. It comes with classes that allow designers to describe the structure of hardware/software systems and a simulator that allows the design to be simulated. This workflow enables the designer to quickly prototype a combined hardware/software system, such as a distributed CPS, without having to decide about particular implementation details early in the design process. These specific details (e.g., whether to implement a particular feature in software or in hardware and what attributes this implementation should focus on) can be omitted when describing a feature in SystemC. This way, this approach offers a more abstract layer on which to outline a system. It reduces the complexity of creating a running prototype by sacrificing implementation details that can be abstracted at an early stage of development, allowing for these details to be added later on once the system has gone through the initial phases of its design.

However, SystemC's C++ foundation also has issues that arise for certain tasks connected to designing systems. Especially, analyzing designs becomes a difficult task. First, the language itself comes in many dialects and offers complex statements that are hard to analyze and sometimes ambiguous or cannot be interpreted before running the program. Second, C++ neither offers a native introspection/reflection framework, nor does it impose many restrictions concerning how memory is handled. This makes retrieving any details of a running program a non-trivial task, which is especially problematic, as a SystemC design needs to run through its elaboration phase in order to be even instantiated.

In order to cope with these issues, different approaches for analyzing SystemC designs have been suggested. As the single-best tool that encompasses all use cases has yet to emerge, each of these approaches has its niche for it to be used. This work gives an overview of several approaches that focus on SystemC analysis.

The rest of this paper is structured as follows: Starting with an overview of SystemC in Section 2, it continues with the description of different approaches to SystemC analysis. The first of these are parsers that extract a design from its source code in Section 3. Afterwards, hybrid approaches that combine a static analysis of the program with an instrumented execution are explored in Section 4, aspect-oriented tools that preprocess a given SystemC program before its compilation in Section 5 and machine learning algorithms that analyze a design merely by simulating it in Section 6. Finally, a conclusion in Section 7 gives an overview of each general idea.

## 2. SystemC

The complexity of hardware is increasing. Not only are more and more parts merged and integrated into so-called system-on-chips, embedded systems are also becoming omnipresent and are starting to interact with each other. These compound computer systems, so-called cyber-physical systems, where several hardware and software systems are interacting with each other and the physical entities with which they can interface, are currently not only the research focus of industry and academia, but already present in today's everyday life.

This increasing complexity of hardware systems (both in terms of complexity of a single system and complexity of the compound system of interacting parts) also results in an increasing complexity of the system design process. These systems still need to be designed in order to work as desired. This obvious prerequisite for CPS development is a crucial one that is quickly becoming a bottleneck during the design process: the ability to handle complexity in the chip manufacturing process increases faster than these complex designs can be designed. This design gap [1] is increasingly turning out to be an Achilles heel of the system design process, especially for the complexity of cyber-physical systems.

The classic approach to hardware design is usually based on synthesizable hardware description languages that can directly be translated into a netlist. Designing systems on this so-called register-transfer level (RTL) also means that for all structures, the designer has to decide how they should be implemented. The decision between hardware and software and each of its implementation options is embedded in this classic design flow, adding to the complexity. Designing systems more abstractly represents an approach to deal with this design gap [1] between the ability to design and the ability to build. Higher abstraction levels overcome the complexity issue by allowing the designer to describe the system's behavior using higher level programming languages (such as C++) without specifying the actual implementation of a given feature. Designing systems on this so-called electronic system level (ESL) results in prototypes that can be executed, but not necessarily synthesized, as not all language elements have a counterpart in hardware. Still, this ability to test an abstract system description as if it were a part of the system without actually implementing the hardware (or even deciding whether this part should be implemented in hardware at all) makes it easy to identify potential problems and to test a system before the time-consuming translation to hardware needs to be done.

**Figure 1:**
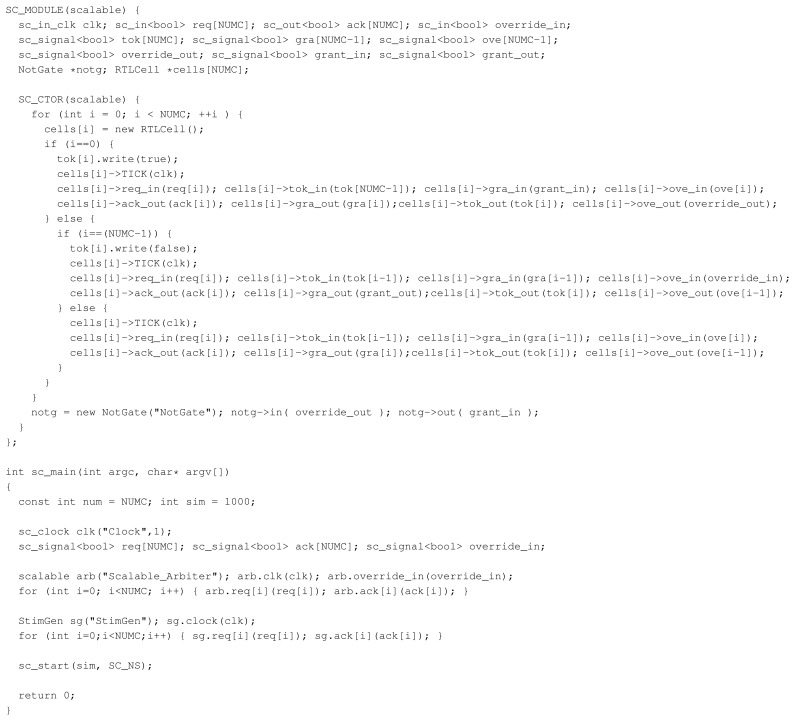
Arbiter example SystemC code. This provides a module scalable that decides which of the NUMC connected devices is granted access to a shared resource (such as a bus or something similar). Notice that a) the constructor SC_CTOR(scalable) that is executed each time a scalable arbiter is created is pure C++ code but highly procedural, generating as many outputs, inputs and RTLCell submodules (which are omitted in this figure) as needed and interconnecting them as required and b) the start of the simulation phase using the sc_start command, splitting the code into the elaboration phase (before starting the simulation) where new objects are created and the simulation phase (after starting the simulation) where the system is run. Figure 2 illustrates the resulting design.

The current *de facto* standard for designing systems on the ESL is SystemC [2]. SystemC is a C++ library, enabling designers to both implement and simulate a system using the library's structures and any C++ construct (that is supported by the compiler being used). A SystemC program consists of two parts, the elaboration phase and the simulation phase. The former is used to setup the design itself: instances of, e.g., modules and signals are created. As this process is also written in C++, it may also be rather dynamic as illustrated in Figure 1: apart from a simple chain of instantiations, more dynamic constructs, such as user input or library calls, may be part of this process. The latter is used to run the resulting system. A simulation kernel invokes the methods of modules that have been triggered by stimuli and, e.g., transfers signals between modules.

Despite the abstract description, designing complex systems in SystemC still results in complex system descriptions. To efficiently design large, complex systems, additional tools beside the integrated development environment (IDE) are usually needed. Visualizations of systems can be used to quickly communicate design features between designers; verification engines check the system for errors with respect to given properties; and the analysis of designs can assist the designer in the design process with tasks, such as debugging.

However, extracting this information from a SytemC implementation is much less straightforward than doing so from an RTL description. Unlike RTL designs, which are static in nature and describe one specific design, ESL designs are set up in the elaboration phase of the program, meaning that the source code is merely a description of how the system should be set up, while the actual (virtual) system itself is only created at runtime.

As C++ does not provide sophisticated introspection and/or reflection capabilities, retrieving these object instances is not trivial. In fact, C++'s paradigm of having the designers get (only) what they pay for results in most meta information about the program currently running being discarded by the compiler. While this results in the binaries being smaller and the running program offering a slightly better performance, it also means that extracting information about running applications, such as an instantiated SystemC design, is a non-trivial task.

Several methods have been proposed to somehow bypass this issue. This work gives an overview of different methods to retrieve information about SystemC designs and highlights both the advantages and disadvantages of the respective ways.

## 3. Parsers

**Figure 3:**
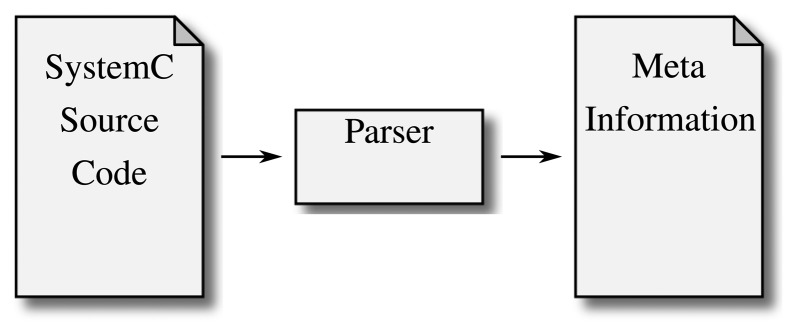
Parsing SystemC: Parsers rely solely on inspecting the source code. While this is a straightforward approach for extracting information and does not rely on a complex architecture, statically analyzing C++ source code is a non-trivial problem.

The first solution to the problem of SystemC design information extraction was to parse the source code and extract the design. Figure 3 illustrates this approach: as the source code does not have to be run, the analysis builds a model from reading the source code alone.

Several dedicated SystemC parsers have been implemented, most of them being open and available for usage.

- ParSyC [3] is a SystemC parser based on the Purdue Compiler Construction Tool Set (PCCTS) [4], the predecessor to Another Tool for Language Recognition (ANTLR) [5]. ParSyC was developed at the University of Bremen, and the source code has not been released publicly yet. ParSyC translates the SystemC description into an abstract syntax tree (AST), builds an intermediate representation from this AST, which can be checked for semantic consistency, and finally synthesizes this intermediate result into a netlist. This last step limits the parser to a synthesizable subset of C++/SystemC, limiting the available code elements to the intersection of the C++ constructs that the parser can understand and those that can be synthesized.
- KaSCPar [6] (Karlsruhe SystemC Parser Suite) is another SystemC parser, created using the Java Compiler Compiler (JavaCC) and the corresponding preprocessor JJTree. As being a user-created parser for SystemC/C++, it has the same problems as ParSyC concerning portability: KaSCPar does not support the whole C++ standard, so compiler-specific additions to the standard library are usually unsupported. The same is true for libraries or anything else that does not have its source code embedded in the project, making many techniques that are a reason for SystemC to have an advantage over RTL development unavailable.
- sc2v [7] (SystemC to Verilog Synthesizable Subset Translator) is a tool that does not primarily analyze a SystemC design, but focuses on the translation from SystemC to Verilog,; however, as a detailed SystemC analysis is a prerequisite to properly translate a given design to Verilog, it fits well into this list. As the primary purpose of this tool is the translation into Verilog, it limits itself to the analysis of a synthesizable subset of SystemC. While this is a reasonable decision for a translation tool, it limits the analysis capabilities.
- SystemCXML [8] uses doxygen [9] as a foundation for its code analysis. Using an existing solution instead of writing a custom C++ parser certainly helps this approach in the analysis process, as C++ constructs are not as limited as a custom solution (that may be incomplete). However, SystemCXML is limited to extracting the properties of the code from the generated doxygen files, basically limiting it to, e.g., a simple list of module instantiations. The conditional creation of modules or, e.g., the interpretation of loops only results in an according syntax tree. SystemCXML does not generate the according structures, limiting its application to, e.g., visualizing the source code instead of the system itself.
- SystemPerl [10] is a collection of Perl scripts that parse SystemC using regular expressions. In order to properly parse the given source code, SystemPerl requires the designer “to provide hints in the program for the preprocessor to identify the constructs to be expanded” [2], meaning that the original source code needs to be modified and prepared for the tool to be able to properly interpret it.
- Another (unnamed) parser, based on using Flex/Bison for lexical and syntactical analysis, was introduced in [11]. This tool relies on processing the code with a C parser after its analysis, which will make it difficult to adopt it to handling the full C++ language.
- Scoot [12] generates a formal model from SystemC designs on which to base its consecutive steps. Just like the other parsers, it relies on a purely static analysis of a given design. In order to make the parsing process more feasible, scoot requires the usage of a customized collection of SystemC header files, which “declare only relevant aspects of the API” [12]. Scoot also uses a modified scheduler that performs better than the standard scheduler that comes with the SystemC kernel. Interestingly, it also provides a way to translate the analyzed design back into a C++ project that does not depend on the SystemC library anymore, resulting (together with the scheduler modifications) in a better simulation performance. However, as scoot only supports a subset of the available SystemC language elements and even modifies the SystemC kernel, utilizing it for an existing project may require some heavy refactoring of the given source code, which might not always be possible.
- systemc-clang [13] is an approach at analyzing SystemC models using clang. Instead of relying on a custom parser, clang is used as a front-end to extract an AST from a given SystemC design. As clang itself provides methods to analyze the AST that it provides, these methods are used to locate and analyze constructs that are specific to SystemC. This approach allows systemc-clang to be a front-end that supports all constructs that clang is able to parse in the first place, thus bypassing most restrictions that come with solutions that employ a custom solution to interpret a given SystemC application. Despite relying on a popular C++ front-end that produces executable binaries if needed, systemc-clang is a purely static approach (hence, its classification as a parser): “The complete architecture of such a [SystemC] model is obtained after the elaboration phase. Hence, systemc-clang extracts the hierarchy of the SystemC model that is available at compile-time only” [13]. Accordingly, systemc-clang offers a comprehensive static code analysis if the program happens to be compatible with the supported dialect, but will fail to analyze designs that rely on dynamic constructs to setup a particular system configuration.

All of these parsers have in common that, by definition, they parse the source code and use this result to extract the system that was described in there.

The advantage of this approach is that the whole analysis process remains in the hand of a single tool. Furthermore, the static approach keeps the architecture simple, as no further execution of a given design is needed in order to extract a synthesizable description.

However, while the static parsing approach works well for designs that describe one single, static system, this approach has some serious drawbacks when it comes to more dynamic structures, such as, e.g., parametrized designs that rely on user input. Parsers, by definition, do not execute a given system. This quickly becomes a problem when taking SystemC's notion of design setup into account where the design itself is created by executing the according C++ code during the elaboration phase. Structures may be created by simply lining up calls to module constructors, which can easily be parsed. However, elaborate designs can be written by creating structures in loops or recursions, creating many instances with just a few lines of code. Creation can also be hidden within macros or other code elements that may or may not be active depending on how the compiler's preprocessor modifies the code. A design may even be created using, e.g., user-defined input values, letting the designer specify, e.g., the number of cores of a simulated CPU or the amount of cache available to a system before running the executable. While complex static structures, such as nested loops, preprocessor directives and compiler-specific language modifications, represent technical issues (*i.e.*, it is hard, but conceptually possible to parse these), parsers are conceptually limited to a purely static approach and, thus, unable to retrieve the correct information if the design relies on information that is not known before executing it. Concerning the example given in Figure 1, this means that parsers will have difficulties analyzing the structure of the arbiter, as the amount of cells within the module might not be fixed. Furthermore, the structure within the module is created using several loops, requiring the parser to go beyond a purely static analysis and, e.g., to execute parts of the code or unroll the loops to extract a model of the design.

To summarize, parsers have serious issues when it comes to parsing complex designs that cannot be solved by more complex parsing techniques. At least the elaboration phase needs to be executed in order to extract a particular system design from a SystemC implementation.

## 4. Hybrid Approaches

Hybrid approaches combine two methods to extract a SystemC design from its source code. First, just like the parsers introduced in Section 3, they apply some kind of static analysis of the source code to extract, e.g., the structure of a given module. Second, however, they solve the problem of analyzing the system's elaboration phase by executing the program at least until the start of the simulation.

However, while the general idea of this approach seems straightforward, extracting information about the structures inside a running C++ program is not easy. As mentioned in Section 2, compilers usually discard all of the information that is not needed to execute a given program. This includes information about the created objects and their structure; in order to execute a C++ program, the program itself does not need the information that an object contains a field called m_internal_state; it just needs to know that a value at address *n* needs to be changed, disregarding even what type the value is at that address.

As the compiler removes the unnecessary information during compilation, it is the one element in the workflow that has access to the information in the first place. Hybrid approaches use this fact to extract the needed data before it is discarded by the compiler by modifying the compilation workflow in a way that lets them extract the information or prevent it from being removed in the first place. Basically, the idea is simple: if the compiler gets rid of the information that is needed, its behavior needs to be modified in order for it to keep it or have it passed on instead.

The compiler needs to analyze the program's static structure anyway in order to compile it. This means that any structural information about the source code is present in the compiler and just needs to be extracted. Any constructs that are usually hard to parse have been properly processed by the time the compiler has analyzed the program structure. Problems therefore either do not arise from the language constructs being used or would also lead to compilation problems when trying to compile the program in order to simulate the system.

The dynamic information (e.g., what modules have been created in the elaboration phase) cannot be extracted at compile time, as the elaboration phase needs to be executed for this information to be present at all. However, as the compiler is the one that translates the program into an executable binary, it can just also modify this binary to track and later store the information about the system being set up.

**Figure 4:**
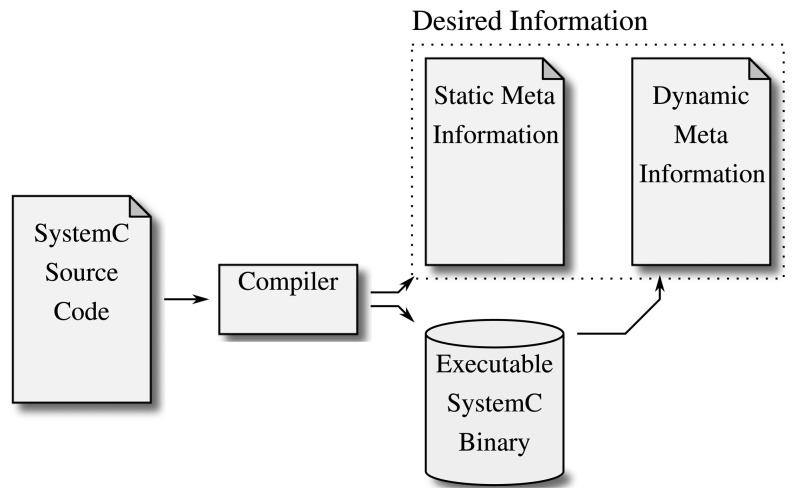
Hybrid approaches split the analysis into two parts. First, the static information is extracted from or by the compiler, which is translating the program anyway. Second, the executable file generated by the compiler is executed, and the dynamic information (esp.SystemC object instances) is extracted from the running program.

Figure 4 shows how this static analysis of, e.g., the program's classes and methods combined with the ability to track the dynamic creation of objects at run-time by modifying the resulting binary file results in a detailed model of the given design. There are a few implementations that follow this idea:

- Pinapa [14] relies on the GNU compiler collection (*GCC*) to analyze SystemC designs. The main advantage of the approach taken is that the limitations of traditional parsers using their own C++ grammars are overcome by utilizing an off-the-shelf front-end instead. This idea, combined with the approach of executing the compiled elaboration phase to retrieve the dynamic instances that describe the actual system, makes Pinapa a much more robust tool than the parsing approaches outlined in Section 3. However, in order to extract the data, it uses a “slightly modified version of SystemC” and requires “a patch to the GNUC++ compiler” [15]. As both the SystemC kernel and the compiler need to be modified for Pinapa to work, this solution is not portable: projects either need to use the given compiler or are simply unsupported. The SystemC kernel modifications restrict projects to the SystemC reference implementation, and even then, problems may arise if the given project needed some modifications to the given SystemC kernel by itself, requiring the two SystemC kernels to be merged. Furthermore, as SystemC itself may be updated, this approach requires constant maintenance to keep up with the changes of the underlying SystemC library.
- A development that improves the approach taken by ParSyC with a dynamic execution of the elaboration phase was also suggested [16]. While this approach solves the parser's problem of not being able to fully extract the elaboration phase's result, the issue of relying on custom parsers for the code interpretation is still present in this approach. Unlike the other dynamic approaches, it does not use an off-the-shelf front-end for C++, thus limiting the available language features, which in turn, might result in the need for serious re-writing of the code.
- PinaVM [17] is the successor of Pinapa. Instead of using a patched GCC, PinaVM relies on using the low level virtual machine (*LLVM*) via its API. Instead of using, e.g., a modified version of GCC (like Pinapa), PinaVM instead relies on using LLVM like a library to handle the given code base. While this has the advantage of being more compatible with version changes (“Although its API is not fully stable, it is clean, and the migration from a version of LLVM to another is a painless task” [17]), it comes at the price of being dependent on what this API offers. As a result of working on LLVM, PinaVM heavily relies on handling the intermediate representation LLVM builds from the source code, the so-called bit code. This assembler-like language is much less abstract than the original C++ code, resulting in each original statement usually being translated into several new statements, which makes the mapping from the used representation to the original source code a non-trivial task. Furthermore, PinaVM, just like Pinapa and SHaBE, relies on one single front-end. As the method itself cannot be transferred to other front-ends, this makes this a powerful, but restricted solution: projects that use code that are incompatible with LLVM are not supported by PinaVM either.
- SHaBE (SystemC Hierarchy and Behavior Extractor) [18] uses a different approach that relies on a debugger instead. While both Pinapa and PinaVM modify the compilation process itself, SHaBE instead uses a combination of the GCC and GNU Project debugger (*GDB*), utilizing the latter to stop the execution of a running SystemC program and to extract the dynamic information from the debugger. Using a predefined set of breakpoints, SHaBE tracks, e.g., the creation of SystemC modules by stopping the execution in the constructor of the sc_module class and inspecting the call stack to get the inheritance hierarchy. At this point, an object's fields can also be retrieved, allowing SHaBE to also extract an object's static features. To retrieve information about the system's behavior, SHaBE goes in a way similar to Pinapa. It hooks into the compiler to retrieve the program's abstract syntax tree, which contains detailed information about the system's functions and their interaction. Instead of modifying the GCC, a plugin is used, allowing SHaBE to be more robust concerning changes of the underlying compiler, assuming that the plugin API does not change frequently. Just like Pinapa and PinaVM, this ties this approach to one specific compiler. As the plugin is not only written for GCC, but other compilers simply do not offer this architecture, it is not portable and relies heavily on the project being interoperable with the chosen architecture. This even excludes projects that rely on workflows based on older versions of the GCC, with the plugin API being a rather recent addition to the project.
- Another approach that uses a debugger is presented in [19]. Although the focus of this approach is on the actual debugging of the system, it still handles the same issues to inspect the system in the first place (*i.e.*, the extraction of the given system's properties). Like SHaBE, this approach uses GDBto extract the data from the running program and therefore suffers the same lock-in issues as the former. While this tool comes with its own visualization to control the debugging environment, it uses a proprietary engine by Concept Engineering to do so, thus limiting the availability of the system.

While the approach differs slightly for these implementations, they share the same features of a dynamic extraction.

Basically, the idea that a compiler has access to all structures and can be used to modify the output in any way holds. A design that is handled using either of these tools can be analyzed well: AST and both static and dynamic structures are extracted, so the output of these methods is thorough.

However, this information retrieval approach does still come with a trade-off. Both solutions are tightly intertwined with the compiler that they are based on (GCC, in both cases); either because the compiler itself is modified (as in Pinapa [14]) or because a plugin specific to that compiler needs to be used during compilation (as in SHaBE [18]). This implies that no other setup may be used in order for the respective implementation to be applied.

The impact of this fact differs depending on the code base. Projects that strictly stick to the C++ standard [20] should be portable enough. As the build process differs from compiler to compiler, setting up the build environment for a new compiler is usually a cumbersome, but still manageable task.

However, C++ comes with dialects and libraries that are not standardized and which jeopardize the application of this approach in other build environments. Different environments have access to different libraries and tools. Microsoft, for example, offers several extensions to the standard C++ library that cannot simply be ported to other build environments [21]. The problem of dialects even exists within the same environment: updating a compiler may break the compilation for some source constructs.

This is a problem for this approach, as the number of available compilers is currently limited, with closed-source compilers probably remaining unsupported, due to the missing ability to add features at will. The solution to rewrite a potentially large code base to get data extraction support is a time-consuming and, therefore, expensive task that should be avoided if possible.

The hybrid approach therefore comes with the most promising, yet also quite limiting notion of either supporting a given SystemC project and being able to export the full design down to the abstract syntax tree or not supporting it at all.

Based on this limitation, we have suggested another approach that sticks to the hybrid approach of separately extracting static and dynamic data from a system, but sets the focus on compatibility in order to support a wider variety of build setups.

- LENSE [22] extracts dynamic and static information without touching a workflow's internals, such as the compiler or the SystemC kernel.
- As compilers need to provide the debugger with the information needed to give a detailed view of the program's state in memory while it is running, they write most of this information to disk during compilation. All major compilers use open formats (such as the STABS[23] or DWARF[24] debug symbols) to store this information or provide an API to access the stored information if it is stored in a proprietary format (such as the PDB format that can be accessed using the debug interface access software development kit (*DIA SDK*) [25].
- If this information is processed instead of the source code itself, the analysis faces far fewer problems than parsers do when analyzing C++, as the pitfalls that are present in C++ code have already been preprocessed by the compiler. Macros, preprocessor directives, includes, *etc.*, have all been properly dealt with, resulting in a pre-analyzed structural description of the program that can more easily be extracted. Furthermore, as the debug symbols (or the respective interfaces) work as an interface between the compiler and the debugger, their structure is unlikely to change often. Combined with the point that all major compilers have accessible symbols, this renders this approach more robust and universally applicable than the modification of one specific compiler.
- To get the dynamic information (e.g., the module instances created during the elaboration phase), a SystemC-API giving the designer access to all SystemC objects can be used. While this API does not give out much information beyond a pointer to all SystemC objects, the combination of this reference with C++'s Run-Time Type Information (RTTI) that retrieves the actual type of a given object and the previously extracted structural information gives the designer much information about a given SystemC object.
- When using the debug information at run-time, the extracted dynamic instances even exceed the SystemC objects that can be retrieved via the SystemC API. As the debug symbols contain the memory layout of a given class (and the objects can be mapped to their classes), references to non-SystemC objects can be resolved at run-time, resulting in a full snapshot of all objects that are referenced by SystemC objects directly or indirectly.
- Compared to other hybrid approaches, the data can be retrieved practically without further prerequisites from any given setup. However, the static data of this approach are limited to the information present in the debug symbols, which usually does not include the full abstract syntax tree. This missing information means that this approach, while being more easily applicable, is best suited for, e.g., design understanding or system analysis purposes. Tasks, like, e.g., verification, that rely on detailed knowledge about a system's behavior are better suited to approaches that return a more detailed model of a given system's internals.

All hybrid methods share one important core feature: the system's elaboration phase is executed, allowing the extraction tool to extract the dynamically generated instances of, e.g., modules or signals. This clearly sets them apart from the static parsing approaches, which rely on being able to statically determine the system's design from its source code, which poses all sorts of problems, including user-generated input, like files, to be read by or parameters to be passed to the program. Furthermore, the structure within the module is created using several loops, requiring the parser to go beyond a purely static analysis and, e.g., execute parts of the code or unroll the loops to extract a model of the design. An ordinary execution of at least the elaboration phase and the successive combination with the static data gathered from, e.g., the source code solves this problem nicely. Concerning the example from Figure 1, the design can be analyzed using hybrid approaches by executing the constructor of the top module and simply monitoring or later extracting all created modules. If the given example contained any platform-specific source code, the major question left would be if the code base is supported by the given tool. However, this approach does not come without certain downsides. First and foremost, the environment used to extract the information needs to support the given code base. This is especially an issue for Pinapa and SHaBE, which are limited to their compiler, with LENSE working on the debug symbols of the major compilers. Second, in the case of LENSE, the user has to pay for the compatibility that the information does not contain, e.g., the full AST.

## 5. Aspect-Oriented Analysis

Especially, Pinapa and SHaBE are tied to their compilers and, hence, are restricted to the input language, which may not match the dialect used in the program to be analyzed. Another method that is based on modifying the source code in a separate preprocessing step before compilation might promise more platform-interoperability while giving more precision than LENSE.

The hybrid approaches outlined in Section 4 rely on modifying the system's dynamic behavior. These modifications are applied during or after compilation. However, a program's behavior might also be modified before compilation by altering the source code instead.

**Figure 5:**
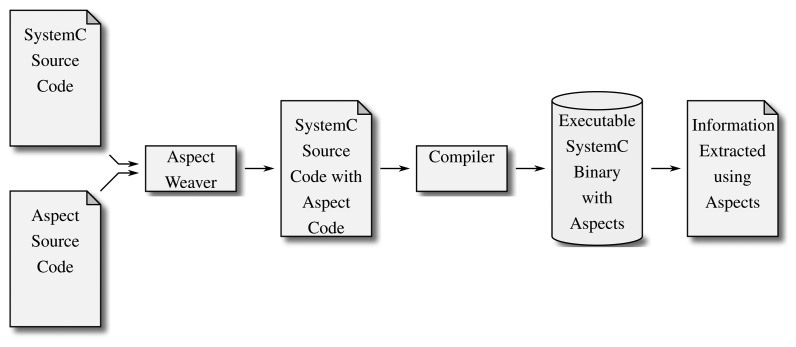
Aspect-oriented programming allows the designer to combine the original SystemC source code with aspect code. A preprocessor (the aspect weaver) combines these into new source code, which is compiled as usual. The aspect code can be used to, e.g., make the resulting binary extract the needed information as needed.

While refactoring a program manually is a tedious, time-consuming task, aspect-oriented programming (AOP) is a paradigm that allows the designer to write refactoring rules that are applied before compiling a program (a process called weaving). Basically, these refactoring rules are separated into elements describing the functionality that is supposed to be inserted into the source code and the definition of locations where these changes are supposed to occur. This scheme therefore allows a designer to describe new functionality (almost) independent of the existing implementation. Figure 5 illustrates how the aspect weaver takes the newly described functionality and inserts it into the existing source code at the defined locations, thereby adding to or replacing functionality that previously existed in the so-called component code with the one described in the additional aspect code.

For C++, there is an open implementation called AspectC++ [26]. AspectC++ has been implemented for all major platforms. The process is fully automatic and can be compared to a preprocessor that modifies the source code before compilation. The major problem with rewriting code to add new functionality (the approach being time consuming and, therefore, expensive) does not apply to this approach, as the refactoring rules are applied automatically.

The aspect weaver is available for both Linux and Windows and generates source constructs that should be compatible with GCC, LLVM/clang and the Microsoft Visual C++ compiler (*MSVC*++), depending on the according setup. The weaver has not yet been ported to encompass the C++11 standard yet. Furthermore, adding it to the compilation workflow of course results in another tool being used that requires the given code base to be compatible. This means that although conceptually, another compatibility issue may be introduced using AOP, AspectC++'s focus on cross-platform usage makes it a much more applicable approach (than, e.g., a hybrid approach using a specific compiler) from a practical point of view.

This approach obviously offers a non-intrusive way to extract information about a running SystemC design, but not just that. As inserting monitor elements that do not alter the way a system works, but merely track what happens while, e.g., the system is simulated is a straightforward application of AOP, several implementations that track SystemC executions have been suggested. However, AOP is not limited to tracking, and other ideas have been built using a combination of AspectC++ and SystemC, as well.

There are several SystemC frameworks or approaches that utilize AOP.

- Feature localization is a way of determining the location of a certain functionality in a design by comparing traces of different runs. Each run is marked to either contain or not contain a certain feature. After executing all runs, the differences in the traces of all runs are used to determine likely locations of each feature.
  In [27], we implemented a feature localization method that uses AOP to embed a gcov-based tracer that traces the executed lines of code into the source code of a given SystemC project in a way that also the object instances (such as modules in a design) that execute a given line are recorded. The resulting feature localization is able to locate features not only in the source code, but is also able to pinpoint object instances that are responsible for triggering these features.
- CHIMP (CHIMP Handles Instrumentation for Monitoring of Properties) [28] is a tool that monitors temporal SystemC properties. The basic approach in this case is that certain properties are hard to describe with standard C++ assertions (especially properties relying on temporal relations), and custom property checkers are hard to embed into existing code bases. AOP, in this case, provides a framework to embed this sophisticated monitor into existing SystemC projects.
- CHIMP adds another layer of abstraction above the usual writing of AOP advice code, taking instead properties and locations in a custom language and generating the according aspect code. Apart from providing a more abstract way to exploit AOP's features, CHIMP also adds functionality beyond the mere translation of its own property expressions into aspects: in order to overcome AspectC++'s limitation of being unable to match arbitrary syntax, CHIMP allows matching any piece of code via regular expressions, as well. While this does not allow matching syntax on a semantic basis (which might be a problem for statements that should be matched, but contain some unexpected character), it is a solution that should work fine for most cases.
- Although not combined in a single tool, [29] outlines the application of AOP for metrics collection, functional verification, communication and the more specific use cases of a cache replacement policy and the separation of control and data streams for given examples.
- Usually, the resulting source code that is generated during the weaving process is quite complex. This is not only a problem for its readability and debugging, but also for the synthesization of hardware from a given SystemC design if the original design was composed from SystemC's synthesizable subset and meant to be translated into hardware. ASystemC [30] is an AOP implementation that, unlike AspectC++, focuses on SystemC and its ties to hardware design. Although the focus on hardware development and synthesizability seems fitting, ASystemC relies on a custom parser for the interpretation of the original SystemC code, sacrificing portability in order to implement its own engine.
- As SystemC designs usually focus on a system's structure and behavior (especially in their early stages of development), concerns, such as power estimation, are usually ignored at first and hard to add later on. AOP provides an easy way to add the needed code for power estimation throughout a design, adding the code that implements the corresponding power model once the structural and functional description is done without the need for any heavy refactoring [31].

The diversity of implemented approaches shows that AOP is well suited to handle many cases in the development of SystemC designs.

There are several advantages to the AOP approach: Aspects can be toggled at will before compilation, resulting in binaries that do not contain code that is not needed. Therefore, there is no performance impact of unused aspects, making them a suitable choice even for expensive operations, as long as rebuilding the project frequently is an option. Furthermore, aspects can be combined. Unlike, e.g., modifications to the SystemC kernel that are not necessarily easy to merge, aspects (at least in theory) can be combined at will. Practically, as refactoring rules usually rely on naming conventions, there might be conflicts or unwanted side-effects when combining several aspects if, e.g., one aspect names a function that results in it being refactored by a second aspect.

Generally, adding a preprocessing step before the compiler's preprocessor is a valid and working approach that solves many problems, but does not come without its penalties.

For example, debugging AOP setups becomes a more complex task. Errors in the aspect code may be inserted into the original code base several hundred times or more, resulting in huge error listings. Furthermore, although AspectC++ adds information for the compiler for which line of the new source code refers to which old one, debugging, e.g., by stepping through the source code does not work well, as the resulting (new) code base contains many constructs that are not easily readable.

Furthermore, AspectC++ currently does not offer the ability to match arbitrary C++ statements. Although [28] added the ability to match any regular expression (which does not necessarily always correspond to a single syntactic or semantic meaning), it is ultimately limited to whatever is available in the source code. Implementing, e.g., a monitor that also tracks calls within a library is therefore not possible using this approach.

Generally, AOP provides an elegant way to alter a given design's source code at compile time to insert missing functionality without excessive manual work. This is especially well suited when the behavior of a given SystemC design should be analyzed, as the aspects are usually focusing on the execution of certain methods. While static analysis might work also by adding static constructs into classes that are then executed due to their presence alone, detailed static analyses, such as the retrieval of a given function's contents in machine-readable form, are not supported by AOP.

## 6. Analysis via Machine Learning

Even the unintrusive approaches that were outlined so far usually have some requirements concerning the project structure or workflow. In order to retrieve more detailed data about the system, most approaches need quite specific setups, which might make it hard to use a certain approach with a particular project.

Another approach to analyze SystemC designs that basically comes without any restrictions at all is one that does not rely on the extraction of additional information concerning the system's structure or behavior, but merely on the observation of the simulation.

Machine learning algorithms have been used to analyze and classify all kinds of systems and information. For system designs, however, their ability to do so purely by observing a given system is a core trait of this approach. Instead of, e.g., analyzing the system's structure, the system's behavior is tracked, and the algorithm attempts to learn as much about the system's structure from this observation.

As SystemC offers an API that gives access to the signals that connect the different modules, this basic observation technique can be used for almost any SystemC design. Most of all, as this approach does not require the data it gets to be complete, no further access to, e.g., the modules' internal structure is needed if it is unavailable.

Of course, if a system does not use a certain functionality, this functionality cannot be recognized by the machine learning algorithm. This makes this approach a very different one, never giving the designer the ability to be sure about the completeness of the given analysis, but at the same time, being extremely applicable, usually not requiring any special preparation in order to be applied.

Basically, using this approach, an artificial intelligence (AI) can regard a system's modules as black boxes. As their contents can never be extracted, their logic can still be reverse-engineered by observing the behavior. An AI that watches a module for some time will eventually be able to extract the module's internal logic from monitoring its inputs and outputs, giving the designer a quick understanding of the module's internals without any need for complex analysis tools.

Machine learning approaches have been used to solve different problems that arise from the difficulties of reliably analyzing SystemC designs.

Especially, the field of coverage-directed test generation (CDG) may benefit from machine learning algorithms as, unlike for, e.g., formal verification methods, incompleteness of the given information still allows the approach to be applied successfully. While for the verification task, fuzzy or incomplete knowledge rules out the ability to make any definite statements, this incomplete amount of information can be communicated to the designer, who can then decide whether to trust a certain piece knowledge or not.

**Figure 6:**
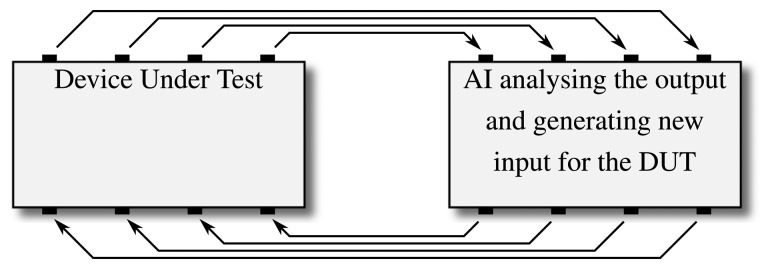
The feedback loop that is implemented for coverage-driven test generation using machine learning algorithms: an AI retrieves the output generated by a given system and generates new input to increase, e.g., a given coverage metric.

A variety of algorithms were implemented to generate test patterns for the simulation phase of a SystemC design. While only a few of them focus on SystemC in particular, the approach of having an algorithm watch the execution is usually portable, allowing for algorithms to be easily adopted to an application on the ESL. The basic function of these approaches is the same throughout: as illustrated in Figure 6, CDG attempts to create a feedback loop where an artificial intelligence analyzes a system's behavior and uses this data to generate stimuli to more quickly achieve a certain coverage goal [32]. Different algorithms have been applied for the step of learning the given system's details. Most works focused on evolutionary algorithms (e.g., [33,34]) or probabilistic models, such as Bayesian Networks or Markov Models (e.g., [35,36]), to learn about the underlying system and generate the according test patterns. The former relies on evolutionary methods to optimize a set of individuals against a certain fitness criterion, which usually corresponds nicely to a given coverage value: individuals (corresponding to a certain set of stimuli) that have already gained better coverage are the ones that are used to generate the next generation of individuals, while those with worse coverage are removed from the pool. The latter relies on probabilistic connections between nodes in directed graphs, modeling the probabilities of how a system changes its state as labels for transitions between a graph's nodes.

While the particular approaches differ concerning, e.g., how detailed the system description has to be for the algorithm to learn from it, the approach of establishing a feedback loop to generate stimuli remains the same for all CDG approaches (see Figure 6).

However, design understanding is not the only field that benefits from an AI analysis.

**Figure 7:**
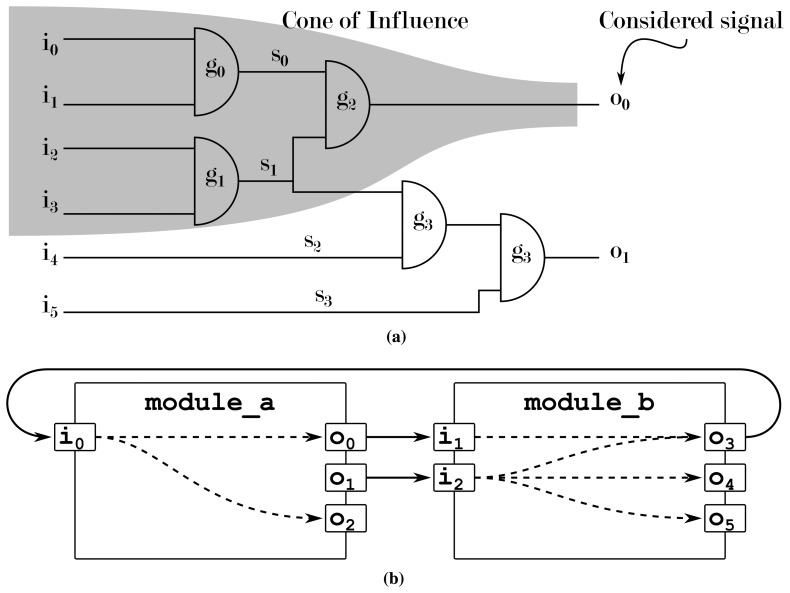
Cone of influence analysis allows a designer to inspect from where, e.g., a faulty signal originated. While this technique is easy to implement on, e.g., the gate level, where full netlists are available, the nature of SystemC's black-box-like modules does not allow a simple extraction of these structures (dotted lines). (**a**) At the RTL or gate level; (**b**) at the ESL.

In [37], we proposed a design understanding approach by using an AI to extract the cone of influence of modules that are otherwise regarded as black boxes. In hardware design, the cone of influence describes how signals influence each other. Based on the visual metaphor of a cone, starting from its tip (a single signal to be observed), a widening cone is drawn through the system, as in Figure 7, encompassing more and more signals with growing distance to the tip that are either responsible or possibly influenced by this signal. These dependencies between signals can, e.g., be used to determine the origin of an erroneous signal by tracing the signal connections towards the input of a chip.

Figure 7a shows the analysis on the RT or gate level: a simple search along all connecting elements reveals all dependencies in the system. Figure 7b shows how modules on the ESL do not give out any information concerning which signal is connected to which other signal: the modules' contents remain hidden, and assuming that each output is dependent on all of a module's inputs is usually an over-approximation of the problem. In this case, an AI can be used to determine the real interconnections from the system's behavior instead. This way, a machine learning module determines the dependencies between signals in order to provide designers with a better understanding of the behavior and/or structure of their system. This use case is especially well suited, as the algorithm being used for this approach (the C4.5 algorithm [38]) generates confidence values that give the designer an indicator as to how reliable a given piece of information is.

The example from Figure 1 would not be parsed or executed with specific tracking code inserted. Instead, this approach works “on top” of the existing design, watching the execution of the design via the ordinary SystemC interfaces. The result is that neither platform-specific code elements are a problem nor the creation of the design during the elaboration phase, resulting in an analysis without any prior requirements of the design at the cost of potentially missing, e.g., dependencies in a cell that defines the arbiter's behavior.

All in all, AI methods provide a straightforward way to analyze a system: if the information cannot be retrieved reliably, it is instead learned from the system's known behavior. This approach is (mostly) fast, can (depending on the use case) be carried out offline, meaning that it does not influence the runtime behavior, except for the tracking of the needed data, and is unintrusive, meaning that the underlying project structure and/or source code does not have to be altered in order for the AI methods to be applied.

The major problem with all AI methods in system design is the incompleteness: there is no guarantee that the extracted information will be complete at all. Any AI-based approach therefore needs to be considered carefully before being applied: On the one hand, it offers a simple way to quickly extract information that is quite helpful for many tasks. On the other hand, depending on the generated stimuli, the information may be quite incomplete, so the reliability is an issue that cannot be fixed easily.

## 7. Conclusions

SystemC analysis has been a research focus for about the last decade. Various tools have been created, each of them with its own advantages and shortcomings.

The major question the designer has to answer before picking a particular approach is which information is needed and how much time may be spent on working with the analysis tool. As the different analysis tools and approaches differ fundamentally, so do the retrieved data.

Generally, the parsing approach has fallen behind, nor has there been any further research activity for the last few years. Conceptually, a purely static approach always has the problem of being unable to offer the results of SystemC's elaboration phase. Furthermore, as most parsers come with their own parsing engine, they are unable to interpret the underlying C++ code without limitations. However, as parsers are usually a single fire-and-forget tool, simply testing whether or not a parser works and utilizing it if it does might still be a viable option.

The other approaches all have their advantages and disadvantages that cannot be clearly compared to each other.

Hybrid approaches offer either a very detailed analysis (Pina* or SHaBE) or broad applicability (LENSE), which makes them the obvious choice for a SystemC analysis issue. AOP approaches, on the other hand, allow simple writing of rules that modify a system's behavior. Not only do they support the insertion of testing and instrumentation code into large projects, they can even be used to alter a given system's structure and behavior automatically, making them a good choice for questions concerning a system's dynamic behavior and possibly enabling the system to cover several use cases by changing it as needed just before compilation. AI approaches, on the other hand, offer not only a sophisticated way to generate stimuli, but also are the least invasive way of inspecting a system's structure, enabling the designer to, e.g., inspect a module's internal connections by analyzing the traced values after running the simulation, completely detaching the analysis tool from the compilation and execution.

However, while the amount of approaches and techniques covers a variety of use cases, it also illustrates that there has yet to arrive a single best solution for all problems concerning SystemC analysis. More specifically, each of the given tracks still offers open questions to be solved. A hybrid approach that is unintrusive, portable and offers a complete model of the system would be the next step for hybrid analysis tools. While several works have been published that focus on AOP solutions for SystemC design issues, the topic is still emerging and offers many open research questions all around. To be able to more quickly try solutions using AOP, AspectC++ itself needs to become more applicable: allowing the designers to specify a wider variety of join points (which describe the points in the source code that should be modified) and a better integration with compilers are urgent topics for the application of AOP on the ESL. For AI solutions, a tighter integration with SystemC would probably be the next research step. Merging machine learning algorithms with the data extraction methods outlined in the other chapters could potentially offer much more data for the AI to learn from, resulting in better performances for the application for which the AI is used. Furthermore, machine learning algorithms that focus on the particular features of system designs (e.g., the fact that systems usually are not behaving nondeterministically and that values might influence results for an indefinite amount of time) have not been developed and compared in detail so far.

All in all, however, a variety of tools has been developed that aid designers in developing systems with SystemC. While there are still open questions left, the development of, e.g., CPSs may benefit from the methods that are already available, allowing designers to more quickly grasp or communicate a system's properties and/or to analyze its behavior.

## Figures and Tables

**Figure 2:**
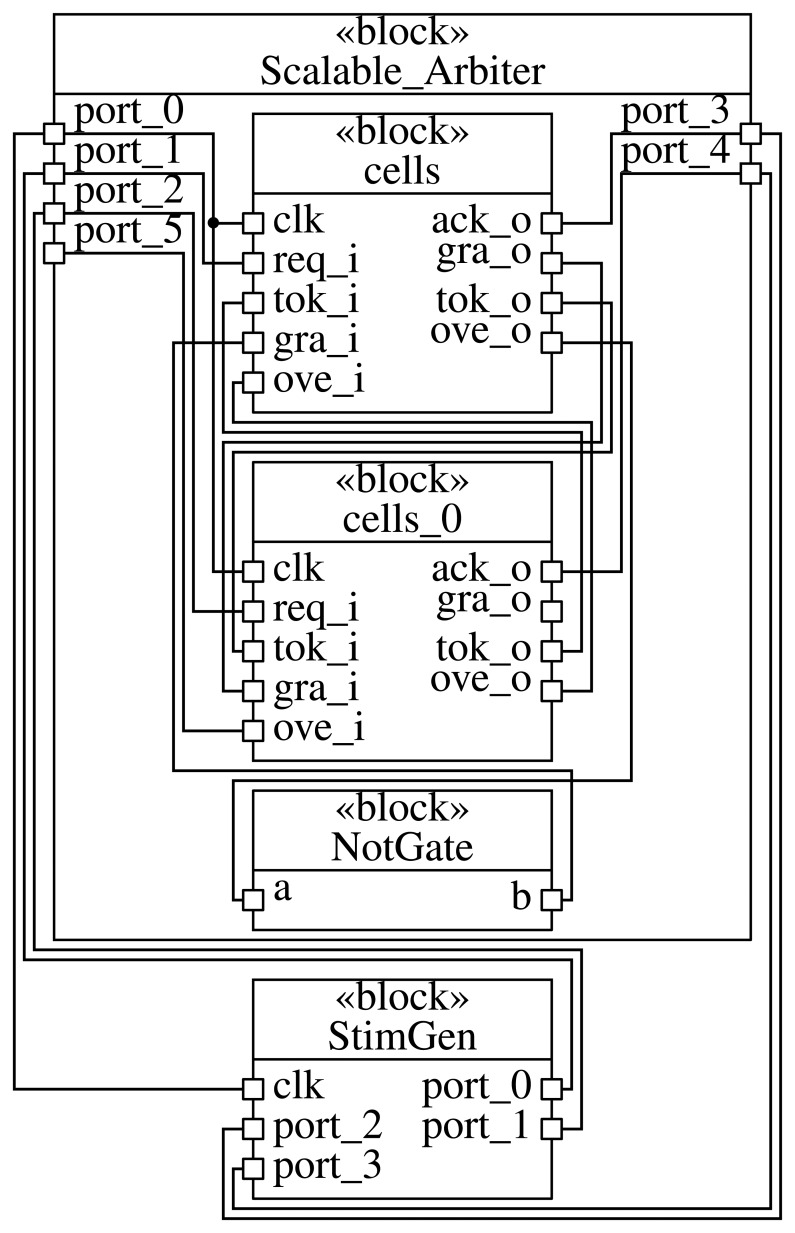
Diagram of the scalable arbiter shown in Figure 1 for a number of cells *NUMC* = 2.
